# *Erratum:* Vol. 65, Suppl. 3

**DOI:** 10.15585/mmwr.mm6602a11

**Published:** 2017-01-20

In the report “Safe and Effective Deployment of Personnel to Support the Ebola Response—West Africa,” which was part of the supplement entitled “CDC’s Response to the 2014–2016 Ebola Pandemic—West Africa and United States,” the following author was omitted from the listing on the first page: **Jacqueline R. Burkholder, PhD, Division of Emergency Operations, Office of Public Health Awareness, CDC.**

